# The impact of cigarette and e-cigarette use history on transition patterns: a longitudinal analysis of the population assessment of tobacco and health (PATH) study, 2013–2015

**DOI:** 10.1186/s12954-020-00386-z

**Published:** 2020-06-29

**Authors:** Lai Wei, Raheema S. Muhammad-Kah, Thaddaeus Hannel, Yezdi B. Pithawalla, Maria Gogova, Simeon Chow, Ryan A. Black

**Affiliations:** 1 Center for Research & Technology, Altria Client Services LLC, 601 East Jackson Street, Richmond, VA 23219 USA; 2 RB Research Consulting Firm Inc., Fort Lauderdale, FL 33312 USA

**Keywords:** Longitudinal transition patterns, Established use behavior, Experimental use behavior, History of cigarette smoking, History of e-cigarette use

## Abstract

**Background:**

Population models have been developed to evaluate the impact of new tobacco products on the overall population. Reliable input parameters such as longitudinal tobacco use transitions are needed to quantify the net population health impact including the number of premature deaths prevented, additional life years, and changes in cigarette smoking prevalence.

**Methods:**

This secondary analysis assessed transition patterns from PATH wave 1 (2013–14) to wave 2 (2014–15) among adult exclusive cigarette smokers, exclusive e-cigarette users, and dual users. Transition probabilities were calculated by taking into account factors including cigarette smoking and e-cigarette use histories and experimental or established use behaviors. Multinomial logistic regression models were constructed to further evaluate factors associated with transition patterns.

**Results:**

Differential transition probabilities emerged among study subgroups when taking into account cigarette smoking and e-cigarette use histories and experimental or established use behaviors. For example, overall 45% of exclusive e-cigarette users in wave 1 continued using e-cigarettes exclusively in wave 2. However, we observed approximately 11 to 14% of wave 1 exclusive experimental e-cigarette users continued to use e-cigarette exclusively in wave 2, compared to about 62% of exclusive established e-cigarette users. The history of cigarette smoking and e-cigarette use is another important factor associated with transition patterns. Among experimental e-cigarette users, 7.5% of individuals without a history of cigarette smoking transitioned to exclusive cigarette smoking, compared to 30% of individuals with a history of cigarette smoking. Additionally, 1.3% of exclusive cigarette smokers in wave 1 transitioned to exclusive e-cigarette use, with the highest transition probability (3.7%) observed in the established cigarette smoker with a history of e-cigarette use subgroup.

**Conclusions:**

Product use histories and current use behaviors are important factors influencing transitions between product use states. Given that experimental users’ transition behaviors may be more variable and more influenced by tobacco use history, long-term predictions made by population models could be improved by the use of transition probabilities from established users. As transition patterns might be changing over time, long-term transition patterns can be examined through analysis of future waves of PATH data.

## Introduction

Prevalence of cigarette smoking among adults in the United States (US) declined from 20.9% in 2005 to 16.8% in 2014 [1], and continues to decline with the most recently reported rate of 14.0% in 2017 [2]. In the meantime, e-cigarettes entered the US market in 2007 and the prevalence among US adults, as reported in the National Health Interview Survey (NHIS), was 2.8% in 2017 [2]. While it is uncertain if emerging tobacco products, such as e-cigarettes, might have contributed to the observed decline in cigarette smoking in recent years [1], population models with appropriate inputs can be useful tools to help understand the potential relationship between e-cigarette uptake and decline of cigarette smoking at the population level.

Adult e-cigarette use patterns have been studied in cross-sectional national surveys, such as NHIS [3–6] and the Behavioral Risk Factor Surveillance System (BRFSS) [7, 8]. However, appropriate population model inputs would ideally benefit from longitudinal studies that measure behavioral transitions over time. The Population Assessment of Tobacco and Health (PATH) study is a nationally representative, longitudinal cohort study of US youth and adults launched in 2011 as a collaborative effort by the US Food and Drug Administration (FDA) Center for Tobacco Products and the National Institutes of Health (NIH) National Institute on Drug Abuse (NIDA). The first wave of data from the PATH study was collected in 2013, and seven waves have been implemented or planned through 2022. The study generates longitudinal epidemiologic data on tobacco use behaviors, including patterns of use, attitudes, beliefs, exposures, and health consequences associated with use of tobacco products among the US population [9]. The study is designed to inform and to monitor the impact of the FDA’s regulatory actions under the Federal Food, Drug, and Cosmetic Act. The longitudinal and comprehensive nature of the PATH datasets provides the opportunity to study cigarette and e-cigarette transition patterns across multiple waves of the PATH study.

There has been a great deal of interest in studying transition patterns between cigarette smoking and e-cigarette use in both youth and adults. Associations between adolescent e-cigarette use and progression to cigarette use, based on PATH wave 1 data, show that among adolescent cigarette experimenters, e-cigarette use was positively and independently associated with progression to current established smoking [10]. Longitudinal associations between non-cigarette tobacco use and subsequent cigarette smoking initiation among US youth indicate that any use of e-cigarettes, hookah, non-cigarette combustible tobacco, or smokeless tobacco is independently associated with cigarette smoking 1 year later and use of more than one product increases the odds of progressing to cigarette use [11]. For adults, an examination of patterns of e-cigarette and cigarette use from PATH wave 1 to wave 2 showed that nearly half (48.8%) of all e-cigarette users in wave 1 had discontinued their use of e-cigarettes in wave 2 [12]. The study also found that among dual users of e-cigarettes and cigarettes at wave 1, 44.3% maintained dual use, 43.5% discontinued e-cigarette use and maintained cigarette smoking, and 12.1% discontinued cigarette use at wave 2. Furthermore, it was concluded that among dual users in wave 1, daily e-cigarette users were more likely than non-daily users to report smoking abstinence in wave 2. A recent study evaluating correlates of transitions in tobacco product use by US adult tobacco users based on data from PATH wave 1 and wave 2 showed that transitions in tobacco product use among adult tobacco users were common overall, but varied among different demographic groups [13]. E-cigarette initiation and associated changes in smoking cessation and reduction were studied among current cigarette smokers aged 25 + years who were not current e-cigarette users in PATH wave 1. The multivariable logistic regression results showed that daily e-cigarette initiators were more likely to have quit smoking cigarettes or reduced use compared to non-users of e-cigarettes, while less frequent e-cigarette use was not associated with cigarette cessation or reduction [14].

Transition probabilities are key inputs for population models to determine the net population health impact of introducing lower-risk non-combustible products such as e-cigarettes into the US market. In recent years, several publications have discussed the use of computational models to assess the overall population level impact of e-cigarettes in terms of changes in smoking prevalence, additional life years, all-cause mortality, smoking-related mortality, etc. [15–21]. Transition probabilities regarding product switching and e-cigarette initiation and cessation were often assumed in published computational models. As an example, a Monte Carlo stochastic simulation model was developed to quantify the balance of health benefits and harms associated with e-cigarette use at the population level [20]. It was concluded that e-cigarette use currently represents more population-level harm than benefit with transition probabilities obtained from multiple cross-sectional national health and tobacco use surveys. However, definitions of product use and quitting behaviors may vary greatly between studies and cross-sectional surveys are not designed to study longitudinal transition patterns, thereby limiting these results. There are other population models [21–26] that have been developed to study the impact of introducing modified risk tobacco products. With reliable input parameters, these models could be used in the future to study the impact of e-cigarettes on the population.

In order to project the long-term impact of e-cigarette use on the US population, transition probabilities representative of the US population are needed to replace assumptions when developing population models. Recently published studies on transition patterns have not distinguished users with different product use histories or experimental/established use behaviors [13]. This secondary data analysis uses PATH wave 1 (2013–2014) and wave 2 (2014–2015) survey data to assess transition patterns among adult cigarette smokers, e-cigarette users, and dual users by taking into account cigarette smoking and e-cigarette use histories and experimental or established use behaviors.

## Methods and analysis

The PATH study is an ongoing, nationally representative, longitudinal cohort study of adults and youth in the USA [9]. Adult respondents in PATH wave 1 were non-institutionalized US civilians aged 18 years and older, while youth respondents were between the ages of 12 and 17 years. Wave 1 data were collected between September 12, 2013 and December 14, 2014, including 32,320 adults (18 + years old) and 13,651 youths^1^. Wave 2 data were collected between October 23, 2014 and October 30, 2015, including 28,362 adults and 12,172 youth. The wave 2 study population consisted of 26,447 adults^2^ who continued from wave 1 (weighted retention rate 83.2%) and 1915 youth respondents from wave 1 who aged up to the adult sample^3^ in wave 2.

To develop transitional patterns among different groups of cigarette and/or e-cigarette users, we analyzed the longitudinal data from the 26,446^4^ adults continuing from wave 1 to wave 2 in the PATH public use data files [27].

Three study groups were defined among wave 1 adult respondents. Group 1 consisted of exclusive cigarette smokers, group 2 consisted of exclusive e-cigarette users, and group 3 included dual users (concurrently using both cigarettes and e-cigarettes).

To evaluate the impact of cigarette smoking and e-cigarette use histories and experimental or established use behaviors, respondents from each group in wave 1 were further categorized into subgroups (Table 1). Exclusive cigarette smokers in wave 1 were categorized into three subgroups: *experimental* cigarette smokers (group 1.1), *established* cigarette smokers *without* a history of e-cigarette use (group 1.2), and *established* cigarette smokers *with* a history of e-cigarette use (group 1.3). Note that we did not have sufficient sample size to further categorize the *experimental* cigarette smokers (group 1.1) based on e-cigarette use history.

**Table 1:** PATH wave 1 definition of user group and subgroup

Wave 1 user group/subgroup	Definition
1. Exclusive cigarette smoker	Currently smokes cigarettes AND does not use e-cigarettes AND may also use tobacco products other than e-cigarettes
1.1. *Experimental* cigarette smoker	Has not smoked 100 or more cigarettes in entire life
1.2. *Established* cigarette smoker *without* a history of e-cigarette use	Has smoked 100 or more cigarettes in entire life AND (has never tried e-cigarettes OR has not used e-cigarettes fairly regularly)
1.3. *Established* cigarette smoker *with* a history of e-cigarette use	Has smoked 100 or more cigarettes in entire life AND (currently does not use e-cigarettes AND has used e-cigarettes fairly regularly)
2. Exclusive e-cigarette user	Currently uses e-cigarettes AND does not smoke cigarettes AND may also use tobacco products other than cigarettes
2.1. *Experimental* e-cigarette user *without* a history of cigarette smoking	Has not used e-cigarettes fairly regularly AND has not smoked 100 or more cigarettes in entire life
2.2. *Experimental* e-cigarette user *with* a history of cigarette smoking	Has not used e-cigarettes fairly regularly AND has smoked 100 or more cigarettes in entire life
2.3. *Established* e-cigarette user *without* a history of cigarette smoking	Has used e-cigarettes fairly regularly AND has not smoked 100 or more cigarettes in entire life
2.4. *Established* e-cigarette user *with* a history of cigarette smoking	Has used e-cigarettes fairly regularly AND has smoked 100 or more cigarettes in entire life
3. Dual user	Currently smokes cigarettes AND uses e-cigarettes AND may also use tobacco products other than e-cigarettes and cigarettes
3.1. *Experimental* dual user^1^	Has not smoked 100 or more cigarettes in entire life OR has not used e-cigarettes fairly regularly^1^
3.2. *Established* dual user	Has smoked 100 or more cigarettes in entire life AND has used e-cigarettes fairly regularly

^1^*Experimental* dual user group include the following subgroups:

(i) Dual user who has not smoked 100 or more cigarettes in entire life AND has not used e-cigarette fairly regularly.

(ii) Dual user who has not smoked 100 or more cigarettes in entire life AND has used e-cigarette fairly regularly.

(iii) Dual user who has smoked 100 or more cigarettes in entire life AND has not used e-cigarette fairly regularly.

Majority of the respondents in the *experimental* dual user group were from group (iii), who were established cigarette smokers and were experimenting e-cigarette use in wave 1. We combined the three subgroups into 3.1. *Experimental* dual user group

Following the same rationale, exclusive e-cigarette users in wave 1 were categorized into four subgroups (Table 1): *experimental* e-cigarette users *without* a history of cigarette smoking (group 2.1), *experimental* e-cigarette users *with* a history of cigarette smoking (group 2.2), *established* e-cigarette users *without* a history of cigarette smoking (group 2.3), and *established* e-cigarette users *with* a history of cigarette smoking (group 2.4).

Dual users in wave 1 were categorized into two subgroups (Table 1): *experimental* dual users (group 3.1) and *established* dual users (group 3.2). The focus of this secondary analysis was to understand transitions among established users in relation to experimental users. Although there could be additional combinations of subgroups in group 3.1 (see footnote 1 in Table 1 for details), for simplicity, we only categorized dual users into two subgroups.

Study group definitions are summarized in Table 1. “Current use” was defined as using the product “every day” or “some days” during the present time. Product use histories and established use behaviors were defined using having “smoked more than 100 cigarettes in entire life” for cigarette smoking and having “used e-cigarettes fairly regularly” for e-cigarette use. These definitions applied in this secondary data analysis are aligned with those employed in the published literature [12, 28, 29].

The definition of e-vapor products varied between PATH wave 1 and wave 2 adult questionnaires. PATH wave 1 focused primarily on “electronic cigarettes, often called e-cigarettes.”. The description of e-cigarettes in wave 1 was as follows, “E-cigarettes look like regular cigarettes, but are battery-powered and produce vapor instead of smoke.” In wave 2, the definition of e-vapor products was broadened to “electronic nicotine products, such as e-cigarettes, e-cigars, e-pipes, e-hookahs, personal vaporizers, vape pens, and hookah pens. Electronic nicotine products are battery-powered, use nicotine fluid rather than tobacco leaves, and produce vapor instead of smoke.” In contrast to wave 1, wave 2 users of each type of electronic nicotine product, such as e-cigarettes, e-cigars, and e-hookahs, were asked about their use patterns. For purposes of calculating transition probabilities, this analysis focused only on e-cigarette data in PATH wave 1 and wave 2 to avoid inflating transition probabilities in favor of the e-cigarette category. There are four product use states based on established cigarette smoking and established e-cigarette use in wave 2 (Table 2). It is important to note that wave 2 use states reflect established product use and may also include users of other tobacco products such as cigars, pipes, or smokeless tobacco. Specifically, the neither group in wave 2 reflects respondents who were not current established cigarette smokers nor current established users of e-cigarettes. By this definition, the wave 2 neither state may include respondents who were current cigarette or e-cigarette experimenters and had not reached the lifetime criteria for cigarettes nor e-cigarettes by wave 2. Thus, the neither state in wave 2 cannot be interpreted as a quitting state and transitions into neither cannot be interpreted as cigarette or e-cigarette cessations.

**Table 2:** PATH wave 2 definition of product use state

Wave 2 product use state	Definition
Exclusive cigarette smoking	A respondent who was a wave 2 established cigarette smoker^a^ AND not a wave 2 established e-cigarette user^b^
Exclusive e-cigarette use	A respondent who was a wave 2 established e-cigarette user^b^ AND not a wave 2 established cigarette smoker^a^
Dual use	A respondent who was a wave 2 established cigarette smoker^a^ AND was also a wave 2 established e-cigarette user^b^
Neither	A respondent who was neither a wave 2 established cigarette smoker^a^ NOR a wave 2 established e-cigarette user^b^

^a^A wave 2 established cigarette smoker is defined as a respondent who was smoking cigarette every day or somedays and had smoked 100 or more cigarettes in entire life by wave 2.

^b^A wave 2 established e-cigarette user is defined as a respondent who was using e-cigarette every day or somedays and had used e-cigarette fairly regularly by wave 2

Statistical analyses were conducted in SAS 9.4. The surveyfreq and surveylogistic procedures were used with wave 2 adult longitudinal weight and replicate weights to adjust for complex survey design, such as oversampling and nonresponse from wave 1 to wave 2. Wave 2 adult longitudinal weights were developed using wave 1 weights as the initial base. The wave 2 weighting process consisted of partitioning the sample into groups defined by wave 1 age, forming weighting classes and performing two nonresponse adjustments, and raking to wave 1 population totals [27]. All estimates were calculated with balanced repeated replication (BRR) using a Fay’s adjustment value of 0.3 based on guidance provided in the PATH user guide [27]. Confidence intervals were computed using the modified Wilson score option.

To assess transition patterns between cigarette smoking and e-cigarette use, we examined changes in self-reported current use state of cigarette smoking and e-cigarette use from wave 1 to wave 2. Wave 1 to wave 2 transition probabilities were calculated for each of the study groups and subgroups as defined in Table 1. For each group or subgroup, there were four possible wave 2 product use states as described in Table 2 (i.e., wave 2 exclusive cigarette smoking, exclusive e-cigarette use, dual use, and neither). We also conducted an age-specific analysis of transition patterns where respondents were grouped into age categories (aged 18–24 years, 25–44 years, or 45–64 years). We did not include respondents older than 65 years of age when calculating age-specific transition probabilities since sample sizes in most exclusive e-cigarette user subgroups were relatively small and we did not observe statistically reliable transition probabilities.

Multinomial logistic regression models were employed to determine factors that may be related to transition patterns between wave 1 and wave 2. Multinomial logistic regression models were fitted to each of the following wave 1 user groups: (1) exclusive cigarette smoker group (model A), (2) exclusive e-cigarette user group (model B), and (3) dual user group (model C).

The outcome variable for wave 2 use state had four levels (i.e., exclusive cigarette smoking, exclusive e-cigarette use, dual use, and neither, as shown in Table 2), where exclusive e-cigarette use was treated as the reference level. We selected wave 2 exclusive e-cigarette use as the reference level because we are specifically interested in comparing product switching behaviors. The following covariates were included in the final models: age (18–24 years, 25–44 years, 45–64 years, 65 + years), gender (male, female), race/ethnicity (non-Hispanic white, non-Hispanic black, non-Hispanic other, Hispanic), education background (less than college, some college, college graduate), poverty level (below poverty level, near poverty level, above poverty level), user subgroups (i.e., defined in Table 1), and years of smoking cigarettes fairly regularly (for respondents who have smoked fairly regularly).

## Results

### Sample composition of adults continuing from wave 1 to wave 2

**Fig. 1:**
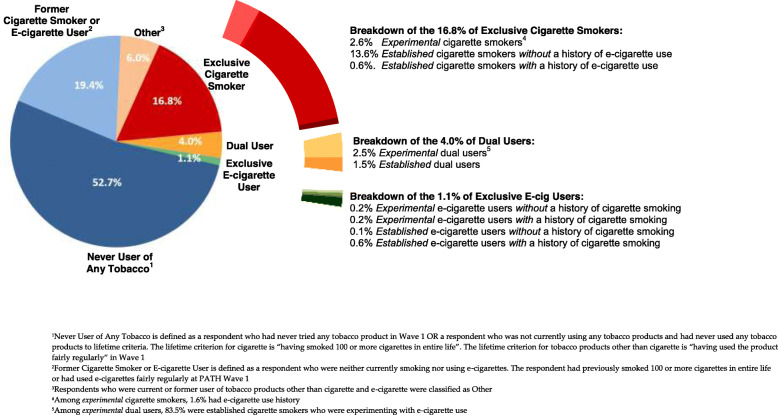
Sample composition of adults continuing from PATH wave 1 to wave 2: cigarette smoking and e-cigarette usage in wave 1 (*n* = 26,446)

The sample composition of 26,446 adults continuing from wave 1 to wave 2 is shown in Fig. 1, with characterization of wave 1 use state. In wave 1, 52.7% were never users of any tobacco, 16.8% were exclusive cigarette smokers, 4.0% were dual users, and 1.1% were exclusive e-cigarette users. Of the 16.8% exclusive cigarette smokers, 2.6% had not smoked 100 cigarettes or more in entire life (*experimental* cigarette smokers), 13.6% were *established* cigarette smokers *without* a history of e-cigarette use, and only 0.6% were *established* cigarette smokers with a history of e-cigarette use. Of the 4.0% dual users, 2.5% were characterized as *experimental* dual users with a majority (83.5%) of *experimental* dual users being *established* cigarette smokers experimenting with e-cigarettes; 1.5% were established dual users. Of the 1.1% exclusive e-cigarette users, 0.4% were *experimental* e-cigarette users, of whom, about half had a history of cigarette smoking; 0.7% were *established* e-cigarette users, 82% of whom had a history of cigarette smoking.

### Transition patterns between cigarette smoking and e-cigarette use

**Fig. 2:**
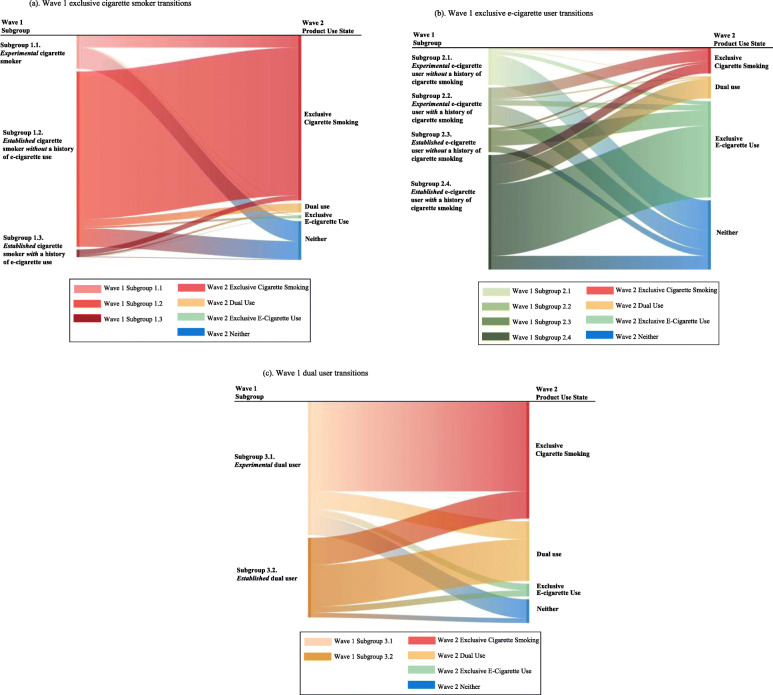
Sankey diagrams by wave 1 study groups based on transitions from wave 1 to wave 2: **(a)** wave 1 exclusive cigarette smoker transitions, **(b)** wave 1 exclusive e-cigarette user transitions and (**c**) wave 1 dual user transitions

Table 3 summarizes overall transition probabilities among the three wave 1 study groups (exclusive cigarette smoker group, exclusive e-cigarette user group, and dual user group), as well as the corresponding subgroups. Age-specific transition probabilities are shown in Tables A.1-A.3 in Supplementary File A. The transitions from wave 1 subgroups to wave 2 product use states are displayed using Sankey diagrams in Fig. 2. The three diagrams in Fig. 2a, b, and c show weighted relative proportions of the subgroups and the corresponding transitions. The transitions are colored by wave 1 subgroups on the left side and the colors change gradually to depict the wave 2 product use states on the right side. In this way, we can understand how wave 1 subgroup populations transitioned into wave 2 where the line weights reflect the transition probabilities in Table 3. Additionally, we can also understand the sources of populations (wave 1 subgroups) by reading from the right side of the figures. As an example from Fig. 2a, we can see majority of wave 1 exclusive cigarette smokers belonged to subgroup 1.2 (*established* cigarette smoker *without* a history of e-cigarette use). The proportion is corresponding to the segments (13.6% out of 16.8%) of the pie chart in Fig. 1. Additionally, majority (corresponding to 84.4% in Table 3) of subgroup 1.2 population stayed as exclusive cigarette smokers in wave 2. When we look at wave 2 exclusive cigarette smoking state, most exclusive cigarette smokers came from subgroup 1.2, followed by subgroup 1.1, and then subgroup 1.3.

**Table 3:** Transition probabilities* from wave 1 to wave 2 with 95% confidence intervals among adults continuing from PATH wave 1 to wave 2 (*n* = 26,446)

Wave 1 study group/subgroup	*n*	Wave 2 product use state			
		Exclusive cigarette smoking % (CI)	Dual use % (CI)	Exclusive e-cigarette use % (CI)	Neither % (CI)
1. Exclusive cigarette smoker	8613	76.4 (75.3, 77.4)	4.3 (3.8, 4.9)	1.3 (1.1, 1.6)	18.0 (17.0, 19.0)
1.1. *Experimental* cigarette smoker	1396	36.7 (34.1, 39.3)	1.3 (0.8, 2.1)	1.8 (1.2, 2.9)	60.2 (57.5, 62.8)
1.2. *Established* cigarette smoker *without* a history of e-cigarette use	6887	84.4 (83.3, 85.4)	4.1 (3.6, 4.7)	1.1 (0.9, 1.4)	10.4 (9.5, 11.4)
1.3. *Established* cigarette smoker *with* a history of e-cigarette use	330	66.4 (60.9, 71.5)	23.1 (18.4, 28.6)	3.7 (2.1, 6.3)	6.8 (4.1, 11.0)
2. Exclusive e-cigarette user	580	12.0 (9.4, 15.4)	10.4 (7.9, 13.5)	45.0 (40.8, 49.3)	32.6 (28.2, 37.2)
2.1. *Experimental* e-cigarette user *without* a history of cigarette smoking	120	7.5^1^ (3.9, 13.9)	- ^2^	10.6^1^ (5.7, 18.7)	80.6 (71.7, 87.2)
2.2. *Experimental* e-cigarette user *with* a history of cigarette smoking	94	30.0 (21.1, 40.7)	- ^2^	14.4 (8.1, 24.3)	51.8 (41.0, 62.4)
2.3 *Established* e-cigarette user *without* a history of cigarette smoking	77	8.2^1^ (3.8, 16.6)	- ^2^	62.2 (49.8, 73.2)	25.9 (15.6, 39.7)
2.4 *Established* e-cigarette user *with* a history of cigarette smoking	289	8.5 (5.8, 12.5)	16.8 (12.7, 21.9)	62.4 (56.7, 67.8)	12.3 (8.3, 17.7)
3. Dual user	2132	54.9 (52.1, 57.6)	28.0 (25.7, 30.3)	6.0 (4.8, 7.5)	11.1 (9.7, 12.7)
3.1 *Experimental* dual user	1340	67.2 (64.1, 70.2)	13.4 (11.6, 15.5)	5.1 (3.8, 6.8)	14.3 (12.3, 16.7)
3.2 *Established* dual user	792	34.3 (30.2, 38.6)	52.3 (48.0, 56.6)	7.6 (5.9, 9.9)	5.8 (4.3, 7.8)

* Records with wave 2 status missing are excluded in the calculation

^1^The estimator is statistically unreliable because the coefficient of variation is greater than or equal to 30 but less than or equal to 50

^2^The estimator is suppressed because there were fewer than 50 total respondents in the group of interest or if coefficient of variation is greater than 50

#### Transitions from group 1: wave 1 exclusive cigarette smoker group

Overall, among exclusive cigarette smokers in wave 1, 76.4% continued to smoke cigarettes exclusively, while 4.3% transitioned to dual use, 1.3% switched to exclusive e-cigarette use, and 18.0% used neither cigarettes nor e-cigarettes in wave 2. Over half (62.0%) of *experimental* cigarette smokers in wave 1 discontinued cigarette smoking in wave 2, with 60.2% using neither and 1.8% transitioning to exclusive e-cigarette use. Among *established* cigarette smokers *without* a history of e-cigarette use, 84.4% continued to smoke cigarettes exclusively in wave 2, while 4.1% transitioned to dual use, 1.1% switched to exclusive e-cigarette use, and 10.4% used neither. Among *established* cigarette smokers *with* a history of e-cigarette use, 66.4% remained as exclusive cigarette smokers in wave 2, which was statistically significantly lower than the 84.4% of *established* cigarette smokers *without* a history of e-cigarette use (*p* < 0.01). *Established* cigarette smokers *with* a history of e-cigarette use had a significantly higher probability of transitioning into dual use compared to *established* cigarette smokers *without* a history of e-cigarette use (23.1% vs. 4.1%, *p* < 0.01). Furthermore, *established* cigarette smokers *with* a history of e-cigarette use had a significantly higher probability of switching to exclusive e-cigarette use compared to those *without* a history of e-cigarette use (3.7% vs. 1.1%, *p* < 0.05).

#### Transitions from group 2: wave 1 exclusive e-cigarette user group

Overall, among exclusive e-cigarette users in wave 1, 45.0% continued to use e-cigarettes exclusively, while 12.0% switched to exclusive cigarette smoking, 10.4% became dual users, and 32.6% used neither product in wave 2.

A majority (80.6%) of *experimental* e-cigarette users *without* a history of cigarette smoking used neither tobacco product in wave 2, compared to 51.8% of *experimental* e-cigarette users *with* a history of cigarette smoking. *Experimental* e-cigarette users *with* a history of cigarette smoking had a significantly higher probability of switching to exclusive cigarette smoking (30.0%) compared to *experimental* e-cigarette users *without* a history of cigarette smoking (7.5%, *p* < 0.01), *established* e-cigarette users *without* a history of cigarette smoking (8.2%, *p* < 0.01), and *established* e-cigarette users *with* a history of cigarette smoking (8.5%, *p* < 0.01).

Among *established* e-cigarette users *without* a history of cigarette smoking, 62.2% continued to use e-cigarettes exclusively in wave 2, while 8.2% switched to smoking cigarettes exclusively. *Established* e-cigarette users *without* a history of cigarette smoking had a significantly higher probability of using neither tobacco product in wave 2 compared to *established* e-cigarette users *with* a history of cigarette smoking (25.9% vs. 12.3%, *p* = 0.04). We also observed a 16.8% transition probability into dual use among *established* e-cigarette users *with* a history of cigarette smoking. The estimates from other subgroups into dual use were suppressed because the coefficients of variation were greater than 50.

#### Transitions from group 3: wave 1 dual user group

Overall, 54.9% of dual users switched to exclusive cigarette smoking. However, the transition patterns between *experimental* and *established* dual user groups were different. *Experimental* dual users had a much higher probability of smoking cigarettes exclusively compared to *established* dual users (67.2% vs. 34.3%, *p* < 0.01). This may be due to the fact that majority (83.5%) of *experimental* dual users are *established* cigarette smokers experimenting with e-cigarette use (see Table 1 footnote). Among *established* dual users, 52.3% continued to dual use, and 7.6% transitioned exclusively to e-cigarette use in wave 2. *Established* dual users had a significantly higher probability of continuing dual use compared to *experimental* dual users (52.3% vs. 13.4%, *p* < 0.01).

### Adjusted odds ratios from multinomial logistic regression models

Table 4 shows adjusted odds ratio (aOR) with 95% confidence intervals from the multinomial logistic regression models. Each model uses one subgroup as the reference group, as listed in the table footnotes. The aORs for all other covariates are shown in Supplementary File B. Unadjusted odds ratios are provided in Supplementary File C.

**Table 4:** Associations of wave 1 cigarette/e-cigarette use state with wave 2 state: adjusted odds ratio* (aOR) with 95% confidence interval (CI)

Model	Wave 1 subgroup	Wave 2 product use state			
		Exclusive cigarette smoking aOR (95% CI)	Dual use aOR (95% CI)	Exclusive e-cigarette use aOR (95% CI)	Neither aOR (95% CI)
Model A^1^. Exclusive cigarette smoker group (*n* = 8613)	1.2. *Established* cigarette smoker *without* a history of e-cigarette use	4.04^‡^ (2.19, 7.45)	4.34^‡^ (1.87, 10.10)	1 [Reference]	0.52^†^ (0.28, 0.97)
	1.3. *Established* cigarette smoker *with* a history of e-cigarette use	1.60 (0.68, 3.74)	9.73^‡^ (3.39, 27.92)	1 [Reference]	0.14^‡^ (0.05, 0.39)
Model B^2^. Exclusive e-cigarette user group (*n* = 580)	2.2. *Experimental* e-cigarette user *with* a history of cigarette smoking	5.36 (0.82, 35.14)	2.63 (0.22, 31.62)	1 [Reference]	0.55 (0.16, 1.87)
	2.3. *Established* e-cigarette user *without* a history of cigarette smoking	0.13^†^ (0.02, 0.87)	0.53 (0.05, 6.25)	1 [Reference]	0.05^‡^ (0.02, 0.17)
	2.4. *Established* e-cigarette user *with* a history of cigarette smoking	0.27 (0.07, 1.06)	2.03 (0.30, 13.84)	1 [Reference]	0.02^‡^ (0.01, 0.06)
Model C^3^. Dual user group (*n* = 2132)	3.2. *Established* dual user	0.30^‡^ (0.20, 0.46)	2.43^‡^ (1.59, 3.73)	1 [Reference]	0.31^‡^ (0.19, 0.51)

*Adjusted odds ratios (aORs) were estimated by multinomial logistic regression models adjusting for age, gender, race/ethnicity, education background, poverty level, user subgroups (i.e., defined in Table 1), and years of smoking cigarettes fairly regularly (for respondents who have smoked fairly regularly).

^1^Model A reference group: group 1.1. *experimental* cigarette smoker

^2^Model B reference group: group 2.1. *experimental* e-cigarette user *without* a history of cigarette smoking

^3^Model C reference group: group 3.1. *experimental* dual user

^†^*p* < 0.05

^‡^*p* < 0.01

#### Model a. wave 1 exclusive cigarette smoker group

Compared to *experimental* cigarette smokers, *established* cigarette smokers *without* a history of e-cigarette use were more likely to either continue smoking cigarettes exclusively (aOR = 4.04, 95% CI 2.19–7.45) or switch to dual use (aOR = 4.34, 95% CI 1.87–10.10) in wave 2 relative to switching to exclusive use of e-cigarettes. Compared to *experimental* cigarette smokers, *established* cigarette smokers *with* a history of e-cigarette use were more likely to transition to dual use relative to switching exclusively to e-cigarettes in wave 2 (aOR = 9.73, 95% CI 3.39–27.92).

Age was found to be a statistically significant covariate in the model (Supplementary File B. Table B1), which is consistent with the different transition probabilities observed among the three different age groups (summary tables in Supplementary File A). In addition to age and education, we found that race may be associated with certain transitions. Other demographic covariates (i.e., gender and poverty level) were not significantly associated with transitioning to cigarette smoking or dual use relative to switching to exclusive e-cigarette use among wave 1 exclusive cigarette smokers.

#### Model B. wave 1 exclusive e-cigarette user group

Compared to *experimental* e-cigarette users *without* a history of cigarette smoking, *established* e-cigarette users had a decreased likelihood of transitioning to exclusive cigarette smoking in wave 2, relative to remaining as exclusive e-cigarette users (aOR = 0.13, 95% CI 0.02–0.87 for those *without* a history of cigarette smoking and aOR = 0.27, 95% CI 0.07–1.06 for those *with* a history of cigarette smoking).

There was statistically significant association between certain age groups (specifically 18–24 age group) and transitions from exclusive wave 1 exclusive e-cigarette use to exclusive cigarette smoking, dual use, or neither, relative to staying in exclusive e-cigarette use (Supplementary File B. Table B2). No statistically significant associations were found between demographic covariates such as gender, education, poverty level, and transition patterns. Race was found to be a statistically significant covariate for certain transitions.

#### Model C. dual user group

Compared to *experimental* dual users, *established* dual users from wave 1 were less likely to switch to exclusive cigarette smoking (aOR = 0.30, 95% CI 0.20–0.46) and were more likely to remain dual users (aOR = 2.43, 95% CI 1.59–3.73) relative to switching to exclusive e-cigarette use in wave 2.

Smoking history (i.e., years of smoking cigarettes fairly regularly) emerged as a statistically significant covariate in the model (Supplementary File B. Table B3), where dual users with more years of cigarette smoking were more likely to transition to exclusive cigarette smoking (aOR = 1.04, 95% CI 1.02–1.07) and dual use (aOR = 1.04, 95% CI 1.01–1.06) relative to switching to exclusive e-cigarette use in wave 2. Other demographic covariates (age, gender, race, education, and poverty level) were not found to be statistically significant.

## Discussion

This secondary analysis of PATH wave 1 and wave 2 study data was performed to evaluate transition patterns between cigarette smoking and e-cigarette use over a 1-year period. Cigarette smoking and e-cigarette use histories along with experimental or established use behaviors were examined as key factors influencing transition patterns. While transitions in tobacco product use among adult tobacco users are common overall [13], this secondary analysis shows that distinct transition probabilities exist among various types of cigarette smokers, e-cigarette users, and dual users.

Exclusive *established* cigarette smokers *without* a history of e-cigarette use were more likely to continue smoking cigarettes, relative to switching to exclusive e-cigarette use. However, among established cigarette smokers with a history of e-cigarette use, we did not observe statistically significant differences in the likelihood of continuing cigarette smoking relative to switching to exclusive e-cigarette use. Exclusive *established* e-cigarette users were less likely to transition to exclusive cigarette smoking, relative to exclusive e-cigarette use. No statistically significant differences in likelihoods were observed for transitioning to dual use, relative to switching to exclusive e-cigarette use across the various exclusive e-cigarette user subgroups. When comparing *established* dual users with *experimental* dual users (a majority of whom were *established* cigarette smokers experimenting with e-cigarette use), *established* dual users were less likely to transition to exclusive cigarette smoking and were more likely to remain dual users, relative to switching to exclusive e-cigarette use.

Differentiated age-specific transition probabilities were observed among individuals aged 18–24 years, 25–44 years, and 45–64 years (Supplementary File A). Furthermore, age emerged as a significant covariate in the multinomial logistic regression models (Supplementary File B). The findings are in line with previous research showing that young adults (aged 18–24 years) are more likely to transition among tobacco products when compared to adults aged 55 + years [13]. Race was also found to be statistically significantly associated with certain transitions, which is consistent with findings from a published study [6].

This secondary data analysis provides realistic transition probabilities, which could be used as input parameters in population models to study the health impact of introducing e-cigarettes into the US population. Instead of making assumptions regarding switching behaviors of cigarette smokers, e-cigarette users, and dual users, the observed transition probabilities provide reliable inputs for population models. In addition, *established* cigarette smokers *with* or *without* a history of e-cigarette use display very different transition probabilities, indicating that subgroups (i.e., those *with* and *without* e-cigarette use history) within the exclusive cigarette smoker population should be considered separately for population models. This rationale can also be applied to the exclusive e-cigarette user population. When modeling e-cigarette user transitions, researchers should consider separating e-cigarette users who were never smokers from e-cigarette users who are former cigarette smokers, as these two groups may have different transition patterns.

Throughout the manuscript, we have made the efforts to clearly define the tobacco user groups and states to support the central analysis objectives. There could be many other ways to define the tobacco user groups and the transition probabilities may vary by different definitions. Studies have shown considerable variations in prevalence estimations with different definitions of current tobacco product use [30, 31]. When studying transitions between two tobacco categories, the complexity increases since we not only need to define the use states for the two categories but also need to consider the usage of tobacco products in other categories. We have chosen to be inclusive so that respondents in the wave 1 subgroups and wave 2 tobacco product use states may use other tobacco products. In addition, although we defined a wave 2 neither state when studying transitions to wave 2, the transition probabilities to the wave 2 neither category cannot be used as cessation rates in population models. The wave 2 neither state may still include respondents who were experimenting with cigarettes smoking or e-cigarette use. Furthermore, as tobacco cessation is often not successful, analyses that were designed to study long-term cessations would be the preferred source for population model inputs [32, 33].

This secondary analysis focused on transitions among adult current tobacco users. Transition probabilities among non-users (e.g., never tobacco users, former users) and youth are also important input parameters for population models. Further analyses can be conducted for both youth and adults to provide comprehensive transition probabilities for population models. In addition to product category level transitions, it is of interest for population models to study transitions at the brand level. Although there are questions around tobacco brands in PATH, there may not be sufficient sample sizes in PATH to conduct brand-specific transition analyses or compare transition patterns across different brands in a certain category.

Given e-cigarettes are relatively new tobacco products within the tobacco space, changes were made in PATH interview questionnaires between waves 1 and 2 to reflect the evolving marketplace. For example, by adding subcategories (i.e., e-cigarette, e-cigars, e-pipes, e-hookahs) to the general electronic nicotine products category in wave 2, about 8.3% (*n* = 245) of PATH wave 1 every day or some days e-cigarette users answered “had never tried electronic nicotine products” in the wave 2 survey and were not able to access questions about e-cigarettes in the wave 2 survey. These respondents were excluded in our secondary analysis, as it appears that they failed to understand that what were referred to as e-cigarettes in wave 1 was a subcategory within the electronic nicotine product category in the wave 2 questionnaire. Furthermore, as the e-cigarette market is evolving and not yet mature, 1-year transition patterns between cigarette smoking and e-cigarette use may change over time. An opportunity to examine transition patterns through multiple waves in order to develop more robust transition probability estimates will be possible as data from future waves of the PATH study become available. Additionally, the calculation of transition probabilities relies on two distinct time points of self-reported “current use” status driven by the design of the PATH study, and does not capture the potential changes that happen between those two time points. In other words, this secondary analysis does not present the entire transition journey individuals have taken during the 12-month period. As an example, an experimental cigarette smoker could have smoked 100 or more cigarettes by wave 2 but was neither smoking cigarettes nor using e-cigarettes every day or some days at the time of the wave 2 interview, which would then be classified as neither a cigarette smoker nor an e-cigarette user in the wave 2 analysis. Lastly, the lifetime use criterion for e-cigarettes, defined as having “used e-cigarettes fairly regularly,” is a qualitative estimate, which may not be fully reliable and subject to interpretation. In comparison, the cigarette lifetime criterion, defined as having “smoked 100 or more cigarettes in entire life” is a well-established quantitative number and is less subject to interpretation.

## Conclusions

In conclusion, 1-year transition patterns differed greatly among various study groups and were driven mostly by age, cigarette smoking and e-cigarette use histories, and experimental or established use behaviors. Overall findings suggest that transition behaviors of experimental users were more variable and more influenced by tobacco use history, while in comparison, transition patterns among established users were more static and less influenced by tobacco use history.

In addition to understanding transition patterns among cigarette smokers, e-cigarette users, and dual users, transition probabilities among established users can be incorporated as input parameters into population models. Results from this secondary analysis are important as they provide realistic transition patterns that can be used to replace assumptions in population models to evaluate the impact of introducing e-cigarettes to the US population. As transition patterns might be changing over time, long-term established transition patterns can be examined through future waves of PATH data.

## Supplementary information

**Additional file 1: Supplementary file A.** Summary of Adult Transition Probabilities by Age.

**Additional file 2: Supplementary file B.** Adjusted Odds Ratios Based on Multinomial Logistic Regression Models.

**Additional file 3: Supplementary file C.** Unadjusted Odds Ratios Based on Multinomial Logistic Regression Models.

## Data Availability

The data analyzed are publicly available and can be downloaded from https://www.icpsr.umich.edu/icpsrweb/NAHDAP/studies/36498.

## Footnotes

1 Detailed sample design, data collection, and response rates can be found in PATH study public use file user guide, https://www.icpsr.umich.edu/icpsrweb/NAHDAP/series/606.

2 Wave 2 adults were adult respondents who completed both wave 1 and wave 2 adult questionnaires.

3 Aged-up adults were youth respondents in wave 1 who had turned 18 years old and agreed to continue in the PATH study as adults in wave 2.

4 There is one respondent who completed both wave 1 and wave 2 questionnaires but the record was removed per respondent request.
