# Supplementary material for: The impact of cigarette and e-cigarette use history on transition patterns: a longitudinal analysis of the population assessment of tobacco and health (PATH) study, 2013–2015
Source: Harm Reduct J. 2020 Jun 29;17:45. doi: 10.1186/s12954-020-00386-z (PMC7322886; doi:10.1186/s12954-020-00386-z)
Supplement: Supplementary file 1 — Additional file 1: Supplementary file A. Summary of Adult Transition Probabilities by Age. [file 12954_2020_386_MOESM1_ESM.docx]

The Impact of Cigarette and E-cigarette Use History on Transition Patterns: A Longitudinal Analysis of the Population Assessment of Tobacco and Health (PATH) Study, 2013-2015

**Supplementary File A**

Lai Wei*^1^, Raheema S. Muhammad-Kah^1^, Thaddaeus Hannel^1^, Yezdi B. Pithawalla^1^, Maria Gogova^1^, Simeon Chow^§1^, and Ryan A. Black^1,2^

^1^Center for Research & Technology, Altria Client Services LLC, 601 East Jackson Street, Richmond, VA 23219, USA; Raheema.S.Muhammad-Kah@altria.com (R.S.M); Thaddaeus.Hannel@altria.com (T.H.); Yezdi.B.Pithawalla@altria.com (Y.B.P.); Maria.Gogova@altria.com (M.G.);

^§^Retired. Sundance@alum.mit.edu (S.C.)

^2^Former Altria Employee. Current Affiliation: RB Research Consulting Firm Inc, Fort Lauderdale, FL 33312, USA; Ryan.Andrea.Black@gmail.com (R.A.B).

*****Correspondence: Lai.Wei@altria.com; Tel.: +1-804-335-3192

Supplementary File A. Summary of adult transition probabilities by age

Table A.1: Summary of Adult Transition Probabilities (%) with 95% Confidence Interval Based on Analysis of Relevant PATH Wave 1 and Wave 2 Data (18-24 Age Group)

| Wave 1 Study Group/Subgroup | n | Wave 2 Product Use State | | | |
| --- | --- | --- | --- | --- | --- |
|  |  | **Exclusive**  Cigarette Smoking  **% (95% CI)** | Dual  Use % (95% CI) | Exclusive E-cigarette Use  **% (95% CI)** | Neither  **% (95% CI)** |
| 1. Exclusive cigarette smoker | 1,875 | 63.2 (60.6, 65.7) | 6.1 (4.9, 7.6) | 2.2 (1.4, 3.3) | 28.5 (25.9, 31.1) |
| 1.1. *Experimental* cigarette smoker | 475 | 21.3 (17.6, 25.5) | 1.51 (0.6, 3.6) | 3.11 (1.7, 5.7) | 74.1 (69.6, 78.1) |
| 1.2. *Established* cigarette smoker *without* a history of e-cigarette use | 1,301 | 77.9 (75.2, 80.3) | 6.4 (4.9, 8.2) | 1.6 (1.0, 2.6) | 14.1 (11.9, 16.7) |
| 1.3. *Established* cigarette smoker *with* a history of e-cigarette use | 99 | 58.6 (48.3, 68.1) | 25.0 (17.5, 34.4) | 5.41 (2.2, 12.3) | 11.01 (5.8, 19.9) |
| 2. Exclusive e-cigarette user | 196 | 16.3 (11.3, 22.8) | 8.4 (5.0, 13.9) | 28.3 (22.4, 35.0) | 47.0 (40.0, 54.2) |
| 2.1. *Experimental* e-cigarette user *without* a history of cigarette smoking | 83 | 9.61 (4.8, 18.2) | -2 | 8.81 (4.1, 18.1) | 79.3 (68.6, 87.1) |
| 2.2. *Experimental* e-cigarette user *with* a history of cigarette smoking | 19 | -2 | -2 | -2 | -2 |
| 2.3 *Established* e-cigarette user *without* a history of cigarette smoking | 48 | -2 | -2 | -2 | -2 |
| 2.4 *Established* e-cigarette user *with* a history of cigarette smoking | 46 | -2 | -2 | -2 | -2 |
| 3. Dual user | 616 | 48.7 (43.9, 53.6) | 27.7 (23.7, 32.0) | 7.8 (5.6, 10.8) | 15.8 (12.6, 19.8) |
| 3.1 *Experimental* dual user | 404 | 57.3 (51.3, 63.1) | 14.1 (11.0, 17.8) | 7.3 (4.9, 10.9) | 21.3 (16.7, 26.7) |
| 3.2 *Established* dual user | 212 | 33.2 (25.6, 41.9) | 52.1 (42.9, 61.1) | 8.6 (5.2, 13.8) | 6.1 (3.5, 10.5) |

^1^The estimator is statistically unreliable because the coefficient of variation is greater than or equal to 30 but less than or equal to 50

^2^The estimator is suppressed because there were fewer than 50 total respondents in the group of interest or if coefficient of variation is greater than 50

Table A.2: Summary of Adult Transition Probabilities (%) with 95% Confidence Interval Based on Analysis of Relevant PATH Wave 1 and Wave 2 Data (25-44 Age Group)

| Wave 1 Study Group/Subgroup | n | Wave 2 Product Use State | | | |
| --- | --- | --- | --- | --- | --- |
|  |  | **Exclusive**  Cigarette Smoking  % (95% CI) | Dual Use  % (95% CI) | Exclusive E-cigarette Use  % (95% CI) | Neither  % (95% CI) |
| 1. Exclusive cigarette smoker | 3,309 | 75.6 (73.8, 77.3) | 5.7 (4.7, 6.8) | 1.6 (1.2, 2.1) | 17.2 (15.6, 18.9) |
| 1.1. *Experimental* cigarette smoker | 408 | 33.3 (28.5, 38.4) | -2 | - 2 | 64.6 (59.5, 69.4) |
| 1.2. *Established* cigarette smoker *without* a history of e-cigarette use | 2,758 | 82.5 (80.6, 84.2) | 5.5 (4.5, 6.7) | 1.6 (1.1, 2.2) | 10.5 (9.1, 12.1) |
| 1.3. *Established* cigarette smoker *with* a history of e-cigarette use | 143 | 66.9 (58.3, 74.5) | 24.7 (17.4, 33.8) | 3.11 (1.2, 7.8) | 5.2^1^ (2.6, 10.4) |
| 2. Exclusive e-cigarette user | 219 | 9.7 (6.5, 14.4) | 13.0 (9.0, 18.4) | 45.9 (39.0, 53.0) | 31.3 (24.7, 38.8) |
| 2.1. *Experimental* e-cigarette user *without* a history of cigarette smoking | 30 | -2 | -2 | -2 | -2 |
| 2.2. *Experimental* e-cigarette user *with* a history of cigarette smoking | 42 | -2 | -2 | -2 | -2 |
| 2.3 *Established* e-cigarette user *without* a history of cigarette smoking | 16 | -2 | -2 | -2 | -2 |
| 2.4 *Established* e-cigarette user *with* a history of cigarette smoking | 131 | 8.1 (4.5, 14.0) | 20.0 (13.7, 28.2) | 62.8 (54.2, 70.6) | 9.2^1^ (5.0, 16.4) |
| 3. Dual user | 884 | 54.6 (51.1, 58.0) | 29.3 (26.0, 32.9) | 5.7 (4.2, 7.9) | 10.3 (8.3, 12.8) |
| 3.1 *Experimental* dual user | 517 | 69.0 (64.4, 73.3) | 13.9 (10.8, 17.7) | 4.2 (2.6, 6.8) | 12.9 (9.8, 16.7) |
| 3.2 *Established* dual user | 367 | 33.8 (29.1, 38.8) | 51.6 (46.4, 56.8) | 8.0 (5.5, 11.4) | 6.6 (4.4, 9.8) |

^1^The estimator is statistically unreliable because the coefficient of variation is greater than or equal to 30 but less than or equal to 50

^2^The estimator is suppressed because there were fewer than 50 total respondents in the group of interest or if coefficient of variation is greater than 50

Table A.3: Summary of Adult Transition Probabilities (%) Based on Analysis of Relevant PATH Wave 1 and Wave 2 Data (45-64 Age Group)

| Wave 1 Study Group/Subgroup | n | Wave 2 Product Use State | | | |
| --- | --- | --- | --- | --- | --- |
|  |  | **Exclusive**  Cigarette Smoking  **% (95% CI)** | Dual  **Use**  % (95% CI) | Exclusive E-cigarette Use  % (95% CI) | Neither  % (95% CI) |
| 1. Exclusive cigarette smoker | 2,836 | 82.2 (80.5, 83.8) | 2.6 (2.1, 3.3) | 0.9 (0.6, 1.3) | 14.3 (12.8, 15.9) |
| 1.1. *Experimental* cigarette smoker | 420 | 48.3 (42.6, 54.1) | 0.9^1^ (0.3, 2.3) | 2.0^1^ (1.0, 4.2) | 48.8 (43.4, 54.3) |
| 1.2. *Established* cigarette smoker *without* a history of e-cigarette use | 2,340 | 88.7 (87.1, 90.2) | 2.4 (1.9, 3.1) | 0.6 (0.3, 1.0) | 8.3 (6.9, 9.9) |
| 1.3. *Established* cigarette smoker *with* a history of e-cigarette use | 76 | 69.0 (56.5, 79.2) | 19.7 (11.6, 31.3) | -^2^ | 7.4^1^ (3.2, 16.1) |
| 2. Exclusive e-cigarette user | 134 | 9.3 (5.2, 16.0) | 7.2^1^ (3.4, 14.6) | 59.6 (50.8, 67.9) | 23.9 (17.0, 32.5) |
| 2.1. *Experimental* e-cigarette user *without* a history of cigarette smoking | 7 | -^2^ | -^2^ | -^2^ | -^2^ |
| 2.2. *Experimental* e-cigarette user *with* a history of cigarette smoking | 24 | -^2^ | -^2^ | -^2^ | -^2^ |
| 2.3 *Established* e-cigarette user *without* a history of cigarette smoking | 11 | -^2^ | -^2^ | -^2^ | -^2^ |
| 2.4 *Established* e-cigarette user *with* a history of cigarette smoking | 92 | -1 | 10.6^1^ (5.1, 20.9) | 71.0 (60.1, 79.9) | 13.7 (7.7, 23.1) |
| 3. Dual user | 560 | 59.7 (54.3, 65.0) | 25.9 (21.9, 30.3) | 5.4 (3.6, 8.0) | 9.0 (6.5, 12.2) |
| 3.1 *Experimental* dual user | 375 | 70.6 (64.3, 76.2) | 12.9 (9.9, 16.7) | 5.0 (3.1, 8.0) | 11.5 (7.9, 16.3) |
| 3.2 *Established* dual user | 185 | 37.0 (28.4, 46.6) | 53.0 (43.5, 62.3) | 6.3^1^ (3.4, 11.4) | 3.7^1^ (1.7, 7.8) |

^1^The estimator is statistically unreliable because the coefficient of variation is greater than or equal to 30 but less than or equal to 50

^2^The estimator is suppressed because there were fewer than 50 total respondents in the group of interest or if coefficient of variation is greater than 50
