# Supplementary material for: The impact of cigarette and e-cigarette use history on transition patterns: a longitudinal analysis of the population assessment of tobacco and health (PATH) study, 2013–2015
Source: Harm Reduct J. 2020 Jun 29;17:45. doi: 10.1186/s12954-020-00386-z (PMC7322886; doi:10.1186/s12954-020-00386-z)
Supplement: Supplementary file 2 — Additional file 2: Supplementary file B. Adjusted Odds Ratios Based on Multinomial Logistic Regression Models. [file 12954_2020_386_MOESM2_ESM.docx]

The Impact of Cigarette and E-cigarette Use History on Transition Patterns: A Longitudinal Analysis of the Population Assessment of Tobacco and Health (PATH) Study, 2013-2015

**Supplementary File B**

Lai Wei*^1^, Raheema S. Muhammad-Kah^1^, Thaddaeus Hannel^1^, Yezdi B. Pithawalla^1^, Maria Gogova^1^, Simeon Chow^§1^, and Ryan A. Black^1,2^

^1^Center for Research & Technology, Altria Client Services LLC, 601 East Jackson Street, Richmond, VA 23219, USA; Raheema.S.Muhammad-Kah@altria.com (R.S.M); Thaddaeus.Hannel@altria.com (T.H.); Yezdi.B.Pithawalla@altria.com (Y.B.P.); Maria.Gogova@altria.com (M.G.);

^§^Retired. Sundance@alum.mit.edu (S.C.)

^2^Former Altria Employee. Current Affiliation: RB Research Consulting Firm Inc, Fort Lauderdale, FL 33312, USA; Ryan.Andrea.Black@gmail.com (R.A.B).

*****Correspondence: Lai.Wei@altria.com; Tel.: +1-804-335-3192

Supplementary file B. Adjusted odds ratios Based on multinomial logistic regression models

**Table B.1. Adjusted Odds Ratio* (aOR) with 95% Confidence Interval (CI) from Model A Wave 1 Exclusive Cigarette Smoker Group (n=8,613)**

| Model Covariate | Wave 2 Product Use State | | | | | | |
| --- | --- | --- | --- | --- | --- | --- | --- |
|  | Exclusive  Cigarette Smoking  aOR (95% CI) | | Dual  Use aOR (95% CI) | | Exclusive E-cigarette Use aOR (95% CI) | Neither  aOR (95% CI) | |
| Age Group = 18-24 | 0.43^†^ | (0.22, 0.85) | 1.00 | (0.39, 2.52) | 1.00 [Reference] | 0.64 | (0.32, 1.29) |
| Age Group = 25-44 | 0.61 | (0.34, 1.10) | 1.27 | (0.65, 2.47) | 1.00 [Reference] | 0.63 | (0.35, 1.14) |
| Age Group = 45-64 | 1.00 | [Reference] | 1.00 | [Reference] | 1.00 [Reference] | 1.00 | [Reference] |
| Age Group = 65+ | 1.51 | (0.20, 11.58) | 1.08 | (0.12, 9.88) | 1.00 [Reference] | 3.09 | (0.43, 22.05) |
| Gender = Male | 0.71 | (0.48, 1.06) | 0.67 | (0.41, 1.10) | 1.00 [Reference] | 0.69 | (0.46, 1.03) |
| Gender = Female | 1.00 | [Reference] | 1.00 | [Reference] | 1.00 [Reference] | 1.00 | [Reference] |
| Race = White Non-Hispanic | 0.50 | (0.25, 1.01) | 1.83 | (0.72, 4.70) | 1.00 [Reference] | 0.44^†^ | (0.22, 0.89) |
| Race = Black Non-Hispanic | 1.00 | [Reference] | 1.00 | [Reference] | 1.00 [Reference] | 1.00 | [Reference] |
| Race = Other Non-Hispanic | 1.00 | (0.33, 3.07) | 4.81^†^ | (1.30, 17.75) | 1.00 [Reference] | 1.14 | (0.37, 3.53) |
| Race = Hispanic | 1.35 | (0.47, 3.93) | 2.49 | (0.69, 9.07) | 1.00 [Reference] | 1.70 | (0.58, 4.96) |
| Education = Less than College | 1.00 | [Reference] | 1.00 | [Reference] | 1.00 [Reference] | 1.00 | [Reference] |
| Education = Some College | 0.60^†^ | (0.38, 0.96) | 0.65 | (0.38, 1.09) | 1.00 [Reference] | 0.83 | (0.51, 1.34) |
| Education = College Graduate | 1.11 | (0.45, 2.74) | 1.12 | (0.42, 3.01) | 1.00 [Reference] | 1.91 | (0.76, 4.81) |
| Poverty level = Below poverty level | 1.00 | [Reference] | 1.00 | [Reference] | 1.00 [Reference] | 1.00 | [Reference] |
| Poverty level = At or near poverty level | 0.69 | (0.42, 1.11) | 0.79 | (0.47, 1.33) | 1.00 [Reference] | 0.78 | (0.47, 1.28) |
| Poverty level = At or above twice poverty level | 0.71 | (0.42, 1.20) | 0.59 | (0.31, 1.10) | 1.00 [Reference] | 1.05 | (0.59, 1.87) |
| Years of smoking cigarettes fairly regularly | 1.01 | (0.99, 1.04) | 1.01 | (0.98, 1.04) | 1.00 [Reference] | 0.98 | (0.95, 1.01) |

* Adjusted odds ratio (aOR) was estimated by adjusting for age, gender, race/ethnicity, education background, poverty level, user subgroups (i.e. defined in Table 1), and years of smoking cigarettes fairly regularly (for respondents who has smoked fairly regularly). ^†^ p < 0.05.
^‡^ p < 0.01.

**Table B.2. Adjusted Odds Ratio* (aOR) with 95% Confidence Interval (CI) from Model B Wave 1 Exclusive E-cigarette User Group (n=580)**

| Model Covariate | Wave 2 Product Use State | | | | | | |
| --- | --- | --- | --- | --- | --- | --- | --- |
|  | Exclusive Cigarette Smoking  aOR (95% CI) | | Dual Use aOR (95% CI) | | Exclusive E-cigarette Use aOR (95% CI) | Neither  aOR (95% CI) | |
| Age Group = 18-24 | 7.13^†^ | (1.32, 38.55) | 8.50^‡^ | (1.97, 36.69) | 1.00 [Reference] | 2.75^†^ | (1.05, 7.21) |
| Age Group = 25-44 | 1.62 | (0.47, 5.65) | 4.52^†^ | (1.26, 16.16) | 1.00 [Reference] | 1.62 | (0.72, 3.65) |
| Age Group = 45-64 | 1.00 | [Reference] | 1.00 | [Reference] | 1.00 [Reference] | 1 | [Reference] |
| Age Group = 65+ | 2.15 | (0.23, 20.21) | 1.83 | (0.26, 12.70) | 1.00 [Reference] | 1.68 | (0.34, 8.40) |
| Gender = Male | 0.93 | (0.38, 2.27) | 1.20 | (0.60, 2.44) | 1.00 [Reference] | 1.25 | (0.74, 2.10) |
| Gender = Female | 1.00 | [Reference] | 1.00 | [Reference] | 1.00 [Reference] | 1 | [Reference] |
| Race = White Non-Hispanic | 0.33^†^ | (0.11, 0.98) | 0.49 | (0.10, 2.32) | 1.00 [Reference] | 0.16^‡^ | (0.05, 0.47) |
| Race = Black Non-Hispanic | 1.00 | [Reference] | 1.00 | [Reference] | 1.00 [Reference] | 1 | [Reference] |
| Race = Other Non-Hispanic | 0.34 | (0.05, 2.26) | 0.16 | (0.02, 1.61) | 1.00 [Reference] | 0.18^†^ | (0.04, 0.89) |
| Race = Hispanic | 0.46 | (0.11,1.88) | 0.12 | (0.01, 1.51) | 1.00 [Reference] | 0.18^‡^ | (0.06, 0.58) |
| Education = Less than College | 1.00 | [Reference] | 1.00 | [Reference] | 1.00 [Reference] | 1 | [Reference] |
| Education = Some College | 0.63 | (0.28, 1.42) | 1.37 | (0.64, 2.91) | 1.00 [Reference] | 1.44 | (0.71, 2.90) |
| Education = College Graduate | 0.76 | (0.26, 2.25) | 2.12 | (0.58, 7.77) | 1.00 [Reference] | 1.44 | (0.56, 3.69) |
| Poverty level = Below poverty level | 1.00 | [Reference] | 1.00 | [Reference] | 1.00 [Reference] | 1 | [Reference] |
| Poverty level = At or near poverty level | 2.34 | (0.82, 6.69) | 0.62 | (0.22, 1.73) | 1.00 [Reference] | 2.63 | (1.00, 6.92) |
| Poverty level = At or above twice poverty level | 2.06 | (0.65, 6.51) | 0.44 | (0.17, 1.16) | 1.00 [Reference] | 1.80 | (0.70, 4.60) |
| Years of smoking cigarettes fairly regularly | 1.02 | (0.97, 1.07) | 1.03 | (0.99, 1.07) | 1.00 [Reference] | 1.02 | (0.99, 1.04) |

* Adjusted odds ratio (aOR) was estimated by adjusting for age, gender, race/ethnicity, education background, poverty level, user subgroups (i.e. defined in Table 1), and years of smoking cigarettes fairly regularly (for respondents who has smoked fairly regularly).

^†^ p < 0.05.
^‡^ p < 0.01.

**Table B.3. Adjusted Odds Ratio* (aOR) with 95% Confidence Interval (CI) from Model C Wave 1 Dual User Group (n=2,132)**

| Model Covariate | Wave 2 Product Use State | | | | | | |
| --- | --- | --- | --- | --- | --- | --- | --- |
|  | Exclusive  Cigarette Smoking aOR (95% CI) | | Dual Use aOR (95% CI) | | Exclusive E-cigarette Use aOR (95% CI) | Neither  aOR (95% CI) | |
| Age Group = 18-24 | 1.73 | (0.81, 3.71) | 1.83 | (0.81, 4.14) | 1.00 [Reference] | 0.90 | (0.35, 2.30) |
| Age Group = 25-44 | 1.97^†^ | (1.12, 3.47) | 1.85 | (0.87, 3.95) | 1.00 [Reference] | 1.08 | (0.50, 2.34) |
| Age Group = 45-64 | 1.00 | [Reference] | 1.00 | [Reference] | 1.00 [Reference] | 1.00 | [Reference] |
| Age Group = 65+ | 0.45 | (0.10, 2.11) | 0.44 | (0.08, 2.42) | 1.00 [Reference] | 2.81 | (0.44, 17.75) |
| Gender = Male | 0.88 | (0.53, 1.46) | 0.89 | (0.54, 1.47) | 1.00 [Reference] | 0.95 | (0.53, 1.70) |
| Gender = Female | 1.00 | [Reference] | 1.00 | [Reference] | 1.00 [Reference] | 1.00 | [Reference] |
| Race = White Non-Hispanic | 1.04 | (0.40, 2.66) | 1.32 | (0.51, 3.41) | 1.00 [Reference] | 0.62 | (0.21, 1.81) |
| Race = Black Non-Hispanic | 1.00 | [Reference] | 1.00 | [Reference] | 1.00 [Reference] | 1.00 | [Reference] |
| Race = Other Non-Hispanic | 1.38 | (0.37, 5.13) | 2.33 | (0.63, 8.63) | 1.00 [Reference] | 1.66 | (0.39, 7.05) |
| Race = Hispanic | 0.53 | (0.19, 1.47) | 0.37 | (0.13,1.05) | 1.00 [Reference] | 0.78 | (0.24, 2.59) |
| Education = Less than College | 1.00 | [Reference] | 1.00 | [Reference] | 1.00 [Reference] | 1.00 | [Reference] |
| Education = Some College | 0.88 | (0.48, 1.62) | 0.90 | (0.49, 1.64) | 1.00 [Reference] | 1.05 | (0.53, 2.05) |
| Education = College Graduate | 1.07 | (0.42, 2.72) | 1.05 | (0.40, 2.77) | 1.00 [Reference] | 2.66^†^ | (1.04, 6.80) |
| Poverty level = Below poverty level | 1.00 | [Reference] | 1.00 | [Reference] | 1.00 [Reference] | 1.00 | [Reference] |
| Poverty level = At or near poverty level | 0.93 | (0.55, 1.59) | 0.94 | (0.55, 1.62) | 1.00 [Reference] | 1.04 | (0.55, 1.98) |
| Poverty level = At or above twice poverty level | 0.80 | (0.41, 1.55) | 0.62 | (0.32, 1.20) | 1.00 [Reference] | 0.97 | (0.50, 1.89) |
| Years of smoking cigarettes fairly regularly | 1.04^‡^ | (1.02, 1.07) | 1.04^‡^ | (1.01, 1.06) | 1.00 [Reference] | 0.98 | (0.94, 1.01) |

* Adjusted odds ratio (aOR) was estimated by adjusting for age, gender, race/ethnicity, education background, poverty level, user subgroups (i.e. defined in Table 1), and years of smoking cigarettes fairly regularly (for respondents who has smoked fairly regularly).

^†^ p < 0.05.
^‡^ p < 0.01.
