# Supplementary material for: The impact of cigarette and e-cigarette use history on transition patterns: a longitudinal analysis of the population assessment of tobacco and health (PATH) study, 2013–2015
Source: Harm Reduct J. 2020 Jun 29;17:45. doi: 10.1186/s12954-020-00386-z (PMC7322886; doi:10.1186/s12954-020-00386-z)
Supplement: Supplementary file 3 — Additional file 3: Supplementary file C. Unadjusted Odds Ratios Based on Multinomial Logistic Regression Models. [file 12954_2020_386_MOESM3_ESM.docx]

The Impact of Cigarette and E-cigarette Use History on Transition Patterns: A Longitudinal Analysis of the Population Assessment of Tobacco and Health (PATH) Study, 2013-2015

**Supplementary File C**

Lai Wei*^1^, Raheema S. Muhammad-Kah^1^, Thaddaeus Hannel^1^, Yezdi B. Pithawalla^1^, Maria Gogova^1^, Simeon Chow^§1^, and Ryan A. Black^1,2^

^1^Center for Research & Technology, Altria Client Services LLC, 601 East Jackson Street, Richmond, VA 23219, USA; Raheema.S.Muhammad-Kah@altria.com (R.S.M); Thaddaeus.Hannel@altria.com (T.H.); Yezdi.B.Pithawalla@altria.com (Y.B.P.); Maria.Gogova@altria.com (M.G.);

^§^Retired. Sundance@alum.mit.edu (S.C.)

^2^Former Altria Employee. Current Affiliation: RB Research Consulting Firm Inc, Fort Lauderdale, FL 33312, USA; Ryan.Andrea.Black@gmail.com (R.A.B).

*****Correspondence: Lai.Wei@altria.com; Tel.: +1-804-335-3192

Supplementary file C. Unadjusted odds ratios (OR) Based on multinomial logistic regression models
Table C. Unadjusted Odds Ratio* (aOR) with 95% Confidence Interval (CI) from Multinomial Logistic Regression Models

| Model | Wave 1 Subgroup | Wave 2 Product Use State | | | | | | |
| --- | --- | --- | --- | --- | --- | --- | --- | --- |
|  |  | Exclusive Cigarette  Smoking OR  (95% CI) | | Dual Use  OR  (95% CI) | | Exclusive E-cigarette  Use OR  (95% CI) | Neither  OR (95% CI) | |
| Model A1.  Exclusive Cigarette Smoker Group  (n=8,613) | 1.2. *Established* cigarette smoker *without* a history of e-cigarette use | 3.68^‡^ | (1.95, 6.95) | 4.83^‡^ | (2.12, 11.00) | 1.00 [Reference] | 0.28^‡^ | (0.15, 0.54) |
|  | 1.3. *Established* cigarette smoker *with* a history of e-cigarette use | 1.15 | (0.54, 2.45) | 9.84^‡^ | (3.66, 26.47) | 1.00 [Reference] | 0.07^‡^ | (0.03, 0.19) |
| Model B2.  Exclusive E-cigarette User Group (n=580) | 2.2. *Experimental* e-cigarette user *with* a history of cigarette smoking | 4.27^†^ | (1.08, 16.82) | 2.64 | (0.28, 25.20) | 1.00 [Reference] | 0.60 | (0.19, 1.87) |
|  | 2.3. *Established* e-cigarette user *without* a history of cigarette smoking | 0.15^†^ | (0.03, 0.82) | 0.56 | (0.06, 5.37) | 1.00 [Reference] | 0.06^‡^ | (0.02, 0.19) |
|  | 2.4. *Established* e-cigarette user *with* a history of cigarette smoking | 0.24^†^ | (0.08, 0.71) | 1.92 | (0.35, 10.36) | 1.00 [Reference] | 0.03^‡^ | (0.01, 0.06) |
| Model C3. Dual User Group  (n=2,132) | 3.2. *Established* dual user | 0.35^‡^ | (0.24, 0.53) | 2.81^‡^ | (1.91, 4.15) | 1.00 [Reference] | 0.28^‡^ | (0.17, 0.46) |

1 Model A reference group: Group 1.1. *Experimental* cigarette smoker

2 Model B reference group: Group 2.1. *Experimental* e-cigarette user *without* a history of cigarette smoking

3 Model C reference group: Group 3.1. *Experimental* dual user

^†^ p < 0.05.
^‡^ p < 0.01.
